# Structural and functional impact of MPro^G48Y/ΔP168^ mutations in SARS-CoV-2 main protease exhibiting resistance to potent inhibitors

**DOI:** 10.1063/4.0000908

**Published:** 2025-10-27

**Authors:** Dipendra Bhandari, Oksana Gerlits, Stephen Keable, Leighton Coates, Annie Aniana, Rodolfo Ghirlando, Nashaat Nashed, Andrey Kovalevsky, John Louis

**Affiliations:** 1 Oak Ridge National Laboratory, Oak Ridge, Tennessee, USA; 2 Tennessee Wesleyan University, Athens, Tennessee, USA; 3 National Institutes of Health, Bethesda, Maryland, USA

## Abstract

Mutations in the main protease (MPro) can alter its enzymatic function and influence drug susceptibility, impacting SARS-CoV-2 maturation and propagation. In this study we examine the effects of G48Y and ΔP168 mutation/deletion, located in the substrate-binding subsites S2 and S4, on dimer stability, enzymatic activity, inhibitor binding, and active site dynamics. Despite these mutations, MPro^G48Y/ΔP168^ retains efficient N- terminal autoprocessing and catalytic activity comparable to the wild type (MPro^WT^). Structural analysis reveals an open active site conformation, increased flexibility in the S2 helix, S5 loop, and helical domain, and a ∼40-fold reduction in dimer dissociation constant (K_dimer_), despite an 8°C decrease in thermal sensitivity to unfolding. Thermodynamic binding studies indicate a 3-fold and 5-fold reduced affinity for nirmatrelvir and ensitrelvir, respectively, while GC373 binding remains unchanged relative to MPro^WT^. Structural comparisons highlighted an open S2 helix and S5 loop conformation in both apo and inhibitor-bound states, suggesting mutational effects are compensated through dynamic adjustments that modulate autoprocessing, dimer stability, and inhibitor interactions. Comparison of MPro^G48Y/ΔP168^ and MPro^WT^ based on B-factor analysis and MD simulations corroborates with enhanced dynamics of the active site and of the entire helical region in the mutant relative to the wild-type enzyme.

